# “I’m Getting Older Too”: Challenges and Benefits Experienced by Very Old Parents and Their Children

**DOI:** 10.1093/geroni/igab046.2781

**Published:** 2021-12-17

**Authors:** Kathrin Boerner, Yijung K. Kim, Elizabeth Gallagher, Kyungmin Kim, Daniela Jopp

**Affiliations:** 1 University of Massachusetts Boston, Boston, Massachusetts, United States; 2 The University of Texas at Austin, Austin, Texas, United States; 3 University of Massachusetts Boston, Boston, MA, Massachusetts, United States; 4 Seoul National University, Seoul, Seoul-t'ukpyolsi, Republic of Korea; 5 University of Lausanne, Lausanne, Vaud, Switzerland

## Abstract

Very old parents and their “old” children are a growing group in industrialized countries worldwide. Since most very old persons have outlived spouses and friends, their children, many of whom have reached old age themselves, are likely to become their primary social contact and to shoulder the care provision role. However, virtually nothing is known about the nature and implications of this relationship constellation. To fill this gap, the present study explored the challenges and rewards of the very old parent-child relationship. In-depth interviews were conducted with 114 parent-child dyads (parent age ≥ 90; child age ≥ 65). Narrative interview data on challenges and rewards were audiotaped, transcribed, and then systematically reviewed and coded, identifying recurrent themes and defining categories that reflected these themes. While both challenges and rewards were present, more rewards than challenges were reported overall. However, comparing parent and child perspectives revealed that the balance of challenges and rewards was less favorable for children. Narrative data further showed that the sense of burdening their children heavily weighed on at least a fourth of parents, reflecting this as a serious concern not only for children but also for parents. Challenges reported by children were often characterized by references to children’s own advanced age and health problems, and the prolonged caregiving involvement due to their parents’ longevity. Healthcare professionals, policy makers, and families should be made aware of this increasingly common phenomenon, and specific services and policies will be needed to adequately support very old adults and their families.

